# Detailed Analysis of Zebrafish Larval Behaviour in the Light Dark Challenge Assay Shows That Diel Hatching Time Determines Individual Variation

**DOI:** 10.3389/fphys.2022.827282

**Published:** 2022-04-11

**Authors:** Sebastian Rock, Frans Rodenburg, Marcel J. M. Schaaf, Christian Tudorache

**Affiliations:** Institute of Biology, Leiden University, Leiden, Netherlands

**Keywords:** diel rhythm, biological clock, stress coping, animal personality, aquaculture

## Abstract

Research on stress coping style, i.e., the response of an organism to adverse conditions, which is constant over time and context, gained momentum in recent years, to better understand behavioural patterns in animal welfare. However, knowledge about the ontogeny of stress coping style is still limited. Here, we performed a detailed analysis of the light dark challenge behavioural assay in zebrafish larvae, where after acclimation in ambient light sudden alternating dark and light phases elicit an anxiety-like response. A principal component analysis on parameters related to locomotion (distance moved, swimming velocity, acceleration, mobility) and directionality (angular velocity, meandering of swimming path) revealed independence between the parameters determined in the light and the dark phases of the assay, indicating unrelated generalised behaviours per phase. However, high collinearity was observed between behavioural parameters within the same phase, indicating a robust response to the stimulus within behavioural phenotypes. Subsequently, this assay was used to determine the correlation between individual hatching time and the behavioural phenotype. The results show that fish that had hatched during daytime have a stronger behavioural response to the dark phase at 5 days post-fertilisation in locomotion related parameters and a weaker response in directionality related parameters, than fish that had hatched during nighttime. These results show that behavioural responses to the light dark challenge assay are robust and can be generalised for the light and the dark phase, and that diel hatching time may determine the behavioural phenotype of an individual.

## Introduction

Consistent individual variation in correlated behavioural and physiological traits, as recognised in coping with stress (Koolhaas et al., 1999, 2010; Korte et al., 2005), is found in virtually all vertebrate taxa, since they are of a huge evolutionary advantage on the population level (Bell, 2007; Øverli et al., 2007; Wolf et al., 2007). Animal welfare research, in particular of farmed animals, benefits immensely from studies of stress coping styles (Castanheira et al., 2017). For example, with the continued growth of the aquaculture industry and increasing scientific discussion over the potential for suffering in farmed fish (Ashley, 2007), individual behavioural and underlying physiological variation in stress coping have been subject of a large number of scientific studies in recent years (Castanheira et al., 2017).

Stress coping styles, i.e., the response of an individual to adverse conditions, which is constant over time and context (Koolhaas et al., 1999; Øverli et al., 2007; Tudorache et al., 2013, 2015, 2018) have often been established through repeatedly measuring anxiety-like behaviour (Koolhaas et al., 1999, 2010; Korte et al., 2005). An often-applied assay for eliciting an anxiety-like response in zebrafish larvae is the light dark challenge, where a zebrafish larva is subjected to a repetition of sudden changes in illumination, with dark phases of several minutes alternating with light phases (Schnörr et al., 2012). In this test, changes in activity levels are triggered by a sudden transition in illumination. A transition from light to dark typically induces an immediate elevated swimming velocity, while a transition from dark to light leads to a sudden reduction of activity (Ellis et al., 2012; Peng et al., 2016; Luchtenburg et al., 2019; Faught and Vijayan, 2021), to a variable extent among different individuals. This test has been used for various studies from basic behavioural research to applied drug screening (e.g., Ellis et al., 2012; Peng et al., 2016; Luchtenburg et al., 2019). The analysis of swimming activity is automated and generates a large number of biomechanical parameters, including locomotion parameters such as swimming speed and acceleration, and directional parameters, such as angular velocity or turning radius. The consistent performance in this test can be used to establish behavioural traits of stress coping styles in larval zebrafish.

However, knowledge about the ontogeny of stress coping styles is still limited and there is a need for early markers (Castanheira et al., 2017). Relationships between stress coping styles of the individual and characteristics expressed in early development, such as the size of the eggs and the yolk reserves (Andersson et al., 2013) and the developmental rate (Andersson and Höglund, 2012), have previously been established. Also, it has been shown in zebrafish that larvae respond to stressors with a similar interindividual variation as adult fish (Tudorache et al., 2013, 2015). These and other developmental manifestations of coping styles were shown to be linked to the time of hatching from the egg or emergence from a spawning nest (Vaz-Serrano et al., 2011; Andersson et al., 2013; Thörnqvist et al., 2015; Leite-Ferreira et al., 2019).

Hatching time of freshwater fish depends on multiple biotic and abiotic factors (Korwin-Kossakowski, 2012) such as pH, oxygen saturation, chemical composition, and salinity (Jezierska, 1988; Oyen et al., 1991; Griem and Martin, 2000; Jezierska and Witeska, 2001; Pyle et al., 2002; Bonisławska, 2010). Temperature, however, exerts possibly the greatest effect on fish development and hatching (Pepin, 1991; Kamler et al., 1994, 1998; Kaminski et al., 2006; Korwin-Kossakowski, 2008). Also the hatching time of zebrafish has been shown to depend on diel temperature and light rhythm (Villamizar et al., 2012). Villamizar et al. (2012) showed that at a constant temperature of 28°C, and a light cycle of 12:12 LD, most larvae hatched around 50 hpf, that is at 2 dpf at ca 4 h after dawn (Villamizar et al., 2012). However, when temperature conditions oscillated between day and night, and/or light conditions were kept constant, peak hatching occurred at later days but always short after dawn (Villamizar et al., 2012). These results indicate a link between hatching time and the biological clock, with light and temperature conditions as external (Zeitgeber) signals.

The biological clock consists in vertebrates of independently operating central and peripheral elements, such as clock genes, oscillating hormone levels, or behavioural activity levels (Takahashi et al., 2008; Tudorache et al., 2018). The biological clock operates autonomously in the absence of external cues, and cycles approximately around a period of 24 h (Takahashi et al., 2008). Ambient external signals, or Zeitgeber, such as light and temperature, affect this internal pattern and generate a diel day-night rhythmicity, which is the base for many essential processes, including locomotor activity, food consumption, sleep, reproductive activity, energy synthesis, hormone production, immune function, or cell-cycle progression (Takahashi et al., 2008).

A link between the activity of the biological clock and individual differences in consistent behaviour have been recognised previously. Individual variation has been observed along the phase of the rhythmic oscillation of the biological clock, resulting in so-called chronotypes which vary in the timing of general activity and biological processes between two extremes, morningness and eveningness, being active early or late during the active phases of the 12-h period, respectively. This phenomenon has been observed in human (Adan et al., 2012) and non-human vertebrates (Randler, 2014). In addition, individual variation in the amplitude of the rhythmic activity has been observed, and has recently been shown to range from a strong rhythm to a near absence of rhythmicity as part of a healthy population in non-human vertebrates (Tudorache et al., 2018). These individual variations in rhythmicity phase and amplitude of the biological clock are correlated to a number of behavioural and physiological traits, such as risk taking or aggressiveness (Maestripieri, 2014; Tudorache et al., 2018), or differences in endocrine baseline levels, e.g., of cortisol (Tudorache et al., 2018), melatonin (Tudorache et al., 2018), or testosterone (Maestripieri, 2014).

In the present study we analyse in detail the light dark challenge behavioural paradigm in larval zebrafish, and show that the performance during a light dark test is correlated with hatching during the dark and the light phase of the diurnal cycle.

## Materials and Methods

### Animal Husbandry

Zebrafish were handled in compliance with the directives of the local animal welfare committee of Leiden University and maintained according to standard protocols.^1^ All protocols adhered to the international guidelines specified by the EU Animal Protection Directive 2010/63/EU. Adult zebrafish were housed and reared in standard Leiden University facility conditions at a constant temperature of 28.5 ± 0.5°C in densities of 40 ± 5 individuals (male:female ∼ 1:1) in 7.5 l tanks in standardised recirculation systems (Fleuren and Nooijen, Nederweert, Netherlands). Ambient light conditions. i.e., 0–425 ± 11 ln m^–2^ with 15 min dusk and dawn period, were maintained as 14:10 h day:night cycles, with light periods 08:00 (0 h Zeitgeber Time, hZT) to 22:00 (14 hZT). Temperature and light conditions are based on standard summer conditions in South Asia, the original geographic range of this species (Engeszer et al., 2007). Fish were fed twice daily, at 1 ± 1 hZT and at 8 ± 1 hZT, with dry food (DuplaRinM, Gelsdorf, Germany) and frozen artemia (Dutch Select Food, Aquadistri BV, Klundert, Netherlands).

**FIGURE 1:**
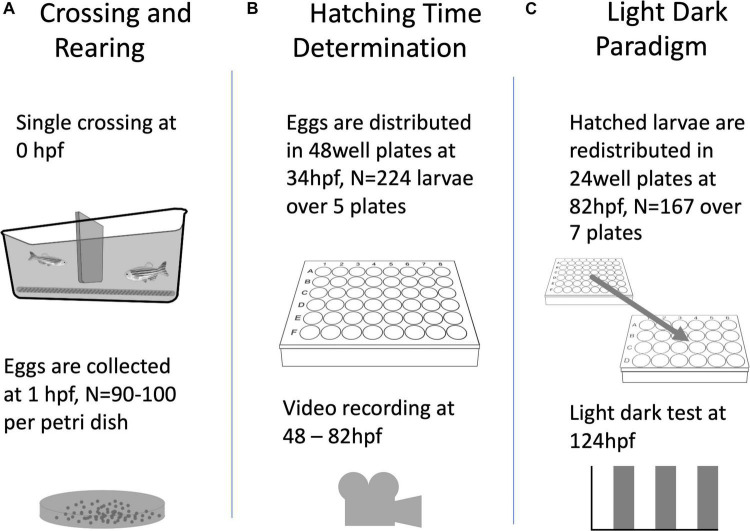
A diagram of the general flow of the experimental procedures described in this paper, including the number of animals used per experiment.

For the generation of larvae (Figure 1A), adult wild type zebrafish (AB/TL) were set up for single pair crossings in tanks (10 × 20 × 10 cm), fit with a spawning mesh and a barrier separating the male from the female. For the single crossing event, the barrier was removed at 09:00, one hour after ambient lights went on (i.e., 1 hZT) and eggs collected 1 h later at 10:00. This ensured minimal variation in fertilisation time while maximising egg production. Five to six pairs of adults were crossed at a time with an average of roughly half of the pairs producing eggs per breeding session. Eggs from all pairs were collected, mixed, and then placed into petri dishes (80–100 per dish) to maximise genetic variation per trial. In-ovo and larval zebrafish were reared in standard rearing conditions: 28.5 ± 0.5°C with a 14/10 h day/night cycle, 80–100 eggs per 90 mm petri dish in egg water (1.2 g Instant Ocean sea salts + 0.25 ml methylene blue per 20 L of milli-Q water) changed daily.

#### Hatching Time

**FIGURE 2:**
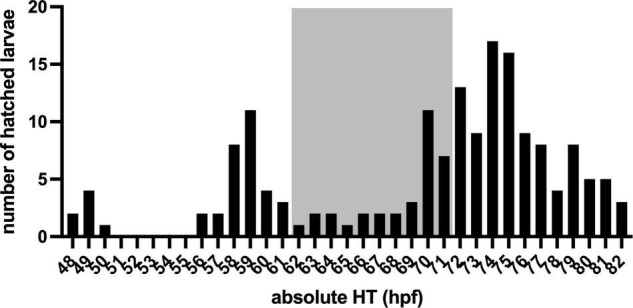
The Histogram of larvae hatched within an observation window between 48 hpf (1 hZT at 2 dpf) and 82 hpf (12 hZT at 3 dpf) in absolute (relative) hatching time (HT, hpf).

To determine hatching time (HT, Figure 1B), the eggs were collected at 34 ± 2 h post-fertilisation (hpf), i.e., 8 ± 2 hZT at 1 day post-fertilisation (dpf) into an egg water filled (1.5 ml per well) 48 well plate with one egg per well. The 48 well plate was placed underneath an infrared (IR) firewire camera (Dragonfly, Point Grey Research Inc., Richmond, Canada), and illuminated with IR light from below. The next day at ca 47 hpf (0 hZT), 10 ml of fresh egg water was added, and a time-lapse movie at 1 frame per 5 min was created with an observation window between 48 hpf (1 hZT at 2 dpf) and 82 hpf (12 hZT at 3 dpf). Subsequently, at 97 ± 1 hpf (2 ± 1 hZT at 4 dpf) and unhatched eggs were removed. Figure 2 shows a distribution histogram of the hatched individuals. Ca 25% of all larvae did not hatch during the observation window. Hatched larvae were subsequently distributed over 24 well plates which were placed on a white background, until behavioural testing commenced at 124 hpf (5 hZT at 5 dpf). This procedure was repeated with 5 plates, resulting in *N* = 224 larvae. HT was determined per full hour by visual observation of the time-lapse movie frames.

### Light Dark Challenge Assay

**FIGURE 3:**
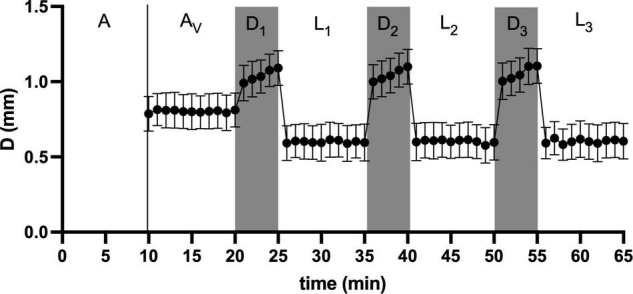
A representative graph of the light dark challenge assay: After an initial acclimation period of 10 min in the illuminated chamber (A), video tracking was initiated for another 10 min (A_*V*_) before the larvae were subjected to a dark challenge phase of 5 min (D_1_) and another light challenge phase of 10 min (L_1_). This challenge chain of dark and light phases was repeated three times (D_2_–L_3_), thus resulting in six alternating dark and light challenge phases. Swimming activity as distance moved (D, mm) typically shows intermediate levels during acclimation, elevated levels during the dark challenge and reduced levels during light challenge levels (mean ± SD, *N* = 152).

We used a repeated light dark challenge assay (Emran et al., 2008; Figure 1C) in order to elicit an anxiety-like response (Steenbergen et al., 2011). At 124 hpf (5 hZT at 5 dpf), the 24 well plate with each well containing a larval zebrafish, was placed in a DanioVision™ observation chamber (Noldus Inc., Wageningen, Netherlands), equipped with IR illumination from beneath the plate and an IR sensitive camera filming from above at 30 fps in a 1280 × 960 pixel resolution. After an initial acclimation period of 10 min in the illuminated chamber, video tracking was initiated for another 10 min before the larvae were subjected to a dark challenge phase of 5 min and another light challenge phase of 10 min. This challenge chain of dark and light phases was repeated three times, thus resulting in six alternating dark and light phases (Figure 3).

### Video Analysis and Data Collection

In order to analyse individual behavioural variation in larval zebrafish, their swimming movements were tracked and a number of parameters for locomotion and directionality were quantified. Larval movement tracking and analysis of the resulting behavioural data was conducted using Ethovision™ software (V14; Noldus, Wageningen, Netherlands). Tracking the centre of mass (CoM) of moving individual larvae over time resulted in a number of behavioural parameters, of which also the standard deviation (SD) was calculated as an estimate for their intra-individual variability. The parameters and their SDs measured per phase were: (a) distance moved (D; mm) and SD(D), i.e., the distance moved by the CoM of individual larvae, averaged over one minute; (b) swimming velocity (V; mm s^–1^) and SD(V), i.e., the average velocity the CoM, averaged over one minute; (c) acceleration (A mm s^–2^) and SD(A), i.e., the changes of V over time, averaged over one minute. These parameter are measures for locomotor activity, and A additionally qualifies changes in swimming velocity with high values indicating high energy expenditure and an erratic swimming mode (Tudorache et al., 2015); (d) meander (M, °mm^–1^) and SD(M), i.e., the amount of change of angular position of the CoM of individual larvae over a distance swum averaged over one minute; (e) angular velocity (Ω; °s^–1^) and SD(Ω) i.e., the rate of change of angular position of the CoM per time interval, averaged over one minute. These last two parameters are measures for the directionality of the swimming path and qualify the swimming mode, with high values indicating an erratic swimming mode (Tudorache et al., 2015); (f) percent immobility (PI;%), i.e., the percent of the observation time spent motionless, as a measure for general activity. Raw data were initially passed through a high-pass (>7.00 mm s^–1^) and a low-pass (<0.02 mm s^–1^) filter of V, in order to filter out erroneously high or low data points, using the Ethovision software. Threshold values were previously determined by approximation from the video images of the swimming larvae, i.e., events of high swimming activity or immobility were verified in the videos, and conservatively estimated. Data were adjusted by calculating the value for M and Ω (adjusted from positive and negative values in the raw data, based on swimming direction right and left), and eliminating negative values for A, since deceleration in swimming larvae is a product of the relatively high viscosity of the water, rather than an active swimming process (Voesenek et al., 2018). All parameters were subsequently calculated as means ± SD over 60 s intervals.

### Behavioural Parameters Interaction

All analyses were conducted in R (version 4.1.0), using the graphical user interface RStudio (RStudio, 2011; R Core Team, 2019). A conditional independence network was constructed by l2-penalised inversion of the covariance matrix, using the rags2ridges package (Van Wieringen and Peeters, 2016). Matrix condition was assessed through a spectral condition number plot (Peeters et al., 2020), and the optimal value for the amount of regularization λ was chosen through leave-one-out cross-validation. The resulting precision matrix was thresholded using a local false discovery rate (lFDR), an approach suggested by Schäfer and Strimmer (2005). A threshold of lFDR < 0.20 was used, as suggested in the original paper (Efron, 2013).

#### Principal Component Analysis

As a quick check, a principal component analysis (PCA, with scaling) was performed using the informative variables for D, SD(D), V, SD(V), M, SD(M), Ω, SD(Ω), and PI (*N* = 167), to see the contribution of each variable to the total variance. In principal components (PC) 1 and 2, containing 65.6% of the total variance, it appears that the variables during the light and dark phase of the light-dark-paradigm act independently of each other, since their loadings are approximately perpendicular. From the plot it can also be seen that there is no obvious pattern of HT across PCs.

#### Partial Correlations

To see which variables directly affect which, a ridge penalised precision matrix was estimated and then thresholded using the local false discovery rate (lFDR). This time we included absolute hatching time (HT, hpf). Partial correlations show the correlation between two variables after accounting for all other correlations those variables have with other variables in the data. If two variables have a partial correlation of 0, then they are conditionally independent. The remaining non-zero partial correlations can be represented by a (weighted) graph. To determine the most influential parameters of the light dark response, we computed two measures of node centrality (betweenness, Eigencentrality) and one measure of node influence (weighted expected force of infection, WE × F). Betweenness is a measure of network centrality. It shows the number of shortest paths between any pair of nodes i and j which pass the node of interest k, where i ≠ j ≠ k. This is divided by the total number of shortest paths (Linton, 1977). Eigencentrality is another measure of network centrality. For a given node i, it corresponds to the number of nodes j ≠ i which themselves have a high Eigencentrality (Landau, 1895). The expected force of infection (E × F), finally is the expected value of the force of infection generated by the node after two transmissions (Lawyer, 2015). We employ a weighted variant (WE × F) that infects adjacent nodes with probability proportional to the strength of the partial correlation (Rodenburg, 2017).

### Correlation Between Behavioural Parameters and Hatching Time

The partial correlation analysis revealed a weak correlation between absolute HT (hpf) and the variation in meandering during the dark phase [SD(M)_*dark*_]. We therefore performed a Spearman rank correlation analysis (*N* = 167, *p* < 0.05) with HT and SD(M)_*dark*_. To visualise the partial correlation between HT and SD(M)_*dark*_, both were regressed on the other variables in the data set and the resulting residuals plotted against each other.

In order to test whether variation in anxiety-like behaviour during the light dark challenge assay is dependent on HT in Zeitgeber time, we plotted behavioural parameters [D, SD(D), V, SD(V), A, SD(A), M, SD(M), Ω, SD(Ω)] for all individual larvae (*N* = 167) averaged over all light and dark phases separately (mean ± SE), over a 24 h range from 0 to 24 hZT, corresponding to 7:00 at 1 dpf to 7:00 at 5 dpf. These sets of values were used for further statistical analyses. All data were subjected to an outlier elimination (ROUT, *Q* = 10%). Data were further checked for normality and homoscedascity using Bartlett’s *t*-test, and the Brown Forsythe test, respectively (significance accepted at *p* < 0.05, *N* = 167). In order to determine the diel HT rhythmicity of locomotor behaviour, a sine wave (non-zero baseline, phase = 24 h) was fitted over the data points of the light and the dark phases per parameter. The resulting amplitudes were then transformed as percentage of the maximum observed value (100%) in order to account for absolute differences between values of the phases of the light dark challenge assay. Subsequently, the percentages were compared using a paired *t*-test (significance accepted at *p* < 0.05, *N* = 167). To determine whether HT at day or night during the 24-h period may have an effect on locomotor parameters of phases, values averaged over the 14:10 h day and night period and light and dark phases were compared (RM Two-way ANOVA with Geisser-Greenhouse corrections, time of the day and phase as factors, Sidack’s *post hoc* test, *p* < 0.05, *N* = 167).

## Results

### Behavioural Parameters Interaction

We determined swimming movement by tracking individual fish over time during the light dark test using automated tracking software, and subsequently analysed the resulting swimming parameters. Parameters for locomotion were distance moved (D; mm), velocity (V; mm s^–1^), acceleration (A; mm s^–2^), and the standard deviations thereof [SD(D), SD(V), and SD(A)]. Parameters for directionality were meandering (M, °mm^–1^) and angular velocity (Ω, °s^–1^) and their standard deviation [SD(M) and SD(Ω)]. A representative graph plotting swimming speed (V in mm s^–1^) over time (s) is presented in Figure 3.

#### Principal Component Analysis

**FIGURE 4:**
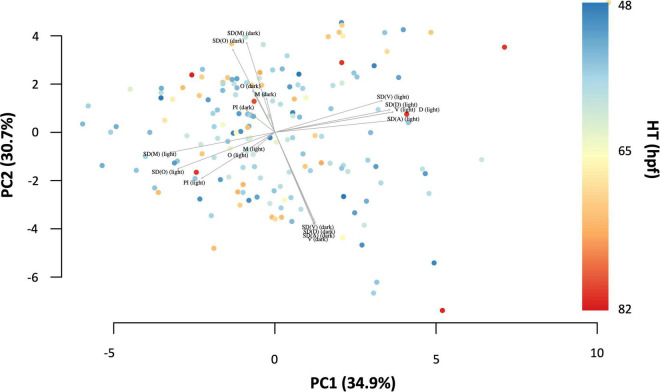
Principal component plot with the first two principal components of the explanatory variables for swimming behaviour during the light dark challenge assay: distance moved (D), velocity (V), acceleration (A), angular velocity (Ω), meandering (M), and percentage immobility (PI) displaying 65.6% of the total variance. Hatching time (HT) is represented by colour.

The principal component analysis (PCA, with scaling) revealed the contribution of each variable to the total variance (Figure 4). The first two principal components, PC1 and PC2, contained together 65.6% of the total variance. It appears as though the variables during light and dark phase of the light dark challenge paradigm act independently of each other, since their loadings are approximately perpendicular. This means that the two clusters can be referred to as Generalised Dark Behaviour (GDB) and Generalised Light Behaviour (GLB).

#### Partial Correlations

**FIGURE 5:**
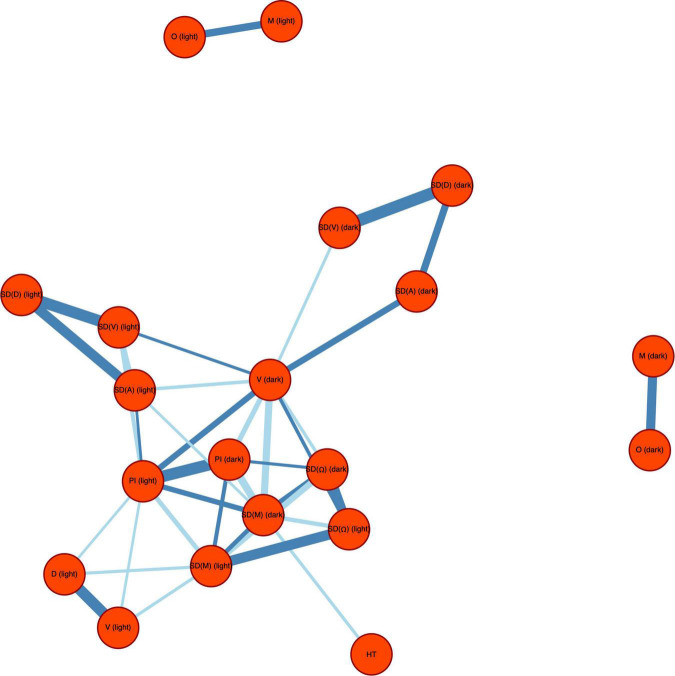
Network of non-zero partial correlations between the explanatory variables for swimming behaviour during the light dark challenge assay: distance moved (D), velocity (V), acceleration (A), angular velocity (Ω), meandering (M), percentage immobility (PI), and hatching time (HT) is represented by colour. The network is thresholded to lFDR < 0.20. Positive partial correlations are indicated by dark blue lines and negative partial correlations by light blue lines. Line width corresponds to the strength of correlation.

The ridge penalised precision matrix, which we performed to detect which variables directly affect which others, revealed strong correlations between related parameters within the locomotion and directionality categories, but not between categories (Figure 5). This indicates a clear separation of parameter categories. In order to identify the parameters which have the strongest expected contribution to the observed values, we measured the network centrality and influence, i.e., a measure of the prominence or importance of an individual factor within a network, by means of betweenness, eigenvector centrality and weighted expected force of infection (WE × F; Table 1). When ranking parameters according to their WE × F value, the parameters SD(M)_*dark*_, PI_*dark*_, PI_*light*_, V_*dark*_, SD(M)_*light*_, SD(Ω)_*dark*_ appear in the top third of the list. Absolute HT (hpf) showed only a weak negative correlation with SD(M)_*dark*_ (Supplementary Figure 1), indicating almost no predictive value for coping style related parameters tested during the light dark assay.

**TABLE 1:** Measures of network centrality and influence for the swimming behaviour parameters during the light and dark phase of the light dark challenge assay: distance moved (D, mm), velocity (V mm s^–1^), acceleration (A mm s^–2^), meandering (M, °mm^–1^), angular velocity (Ω, °s^–1^), and percentage Immobility (PI, %), and the variation thereof in standard deviation (SD).

Parameter	Betweenness	Eigenvector centrality	WE × F
SD(M)_dark_	18.67	1	5.26
PI_light_	21.87	0.96	4.91
PI_dark_	1.05	0.79	4.87
V_dark_	43.57	0.97	4.8
SD(M)_light_	7.83	0.85	4.19
SD(Ω)_dark_	1.05	0.74	4.04
SD(Ω)_light_	0.8	0.62	3.68
SD(A)_light_	8.77	0.63	2.66
SD(V)_light_	4.9	0.48	2.27
SD(A)_dark_	6.5	0.18	1.51
D_light_	0	0.38	1.31
V_light_	0	0.38	1.31
SD(D)_light_	0	0.19	1.3
SD(V)_dark_	6.5	0.18	0.98
HT	0	0.17	0.61
SD(D)_dark_	0.5	0.06	0.4
M_dark_	0	0	0
Ω_dark_	0	0	0
M_light_	0	0	0
Ω_light_	0	0	0

*This table is ranked by WE × F (weighted expected force of infection).*

### Correlation Between Behavioural Parameters and Hatching Time

Since absolute HT (hpf) showed only a weak negative correlation with SD(M)_*dark*_ (Supplementary Figure 1), we transformed absolute HT into relative HT in Zeitgeber time (hZT) by cyclically recalculating it from the first hour of light (0 hZT) to the last hour of darkness (23 hZT), in order to assess whether diel rhythmicity hatching can predict swimming behaviour during the light dark assay. We fitted a sine wave (non-zero baseline, 24-h period) over the relative HT (hZT) data (Supplementary Figure 2), and subsequently percentage transformed the resulting amplitudes in order to compared them a paired *t*-test with multiple testing correction (Bejamini–Hochberg, Supplementary Figure 3). *T*-test variables are given in Table 2. Only meandering (M) and angular velocity (Ω), both locomotion path parameters, showed significantly higher amplitudes for the dark phase values when compared to light phase values (paired *t*-test, M: *N* = 168, *p* = 0.0378; Ω: *N* = 170, *p* < 0.0001, before correction). In order to determine whether the light conditions under which the larvae were hatched may influence locomotion parameters during the phases of the light dark assay, we averaged the movement parameter values of larvae hatched during daytime (i.e., 0–14 hZT) and nighttime (i.e., 15–24 hZT). After confirming normality and homoscedascity of the data (*p* > 0.05), we compared them using a Repeated Measure (RM) Two-way ANOVA with Geisser-Greenhouse Corrections, with phase of the light dark challenge (i.e., light or dark), time of day when hatching occurred (i.e., daytime or nighttime) and interactions as factors. An RM approach was accounting for the fact that the same fish were measured during the different phases of the challenge. For all locomotion parameters and their variations, only the phase of the light dark assay showed a significant effect, with the exception of A, for which also time of day had an effect on the differences observed (Table 3). The results for the parameters describing locomotion path were mixed: For both M and Ω, the effect of time of day were significant, but not the effect of phase, while the other parameters showed a significant effect of phase but not of time of day (Table 3).

**TABLE 2:** Statistical details for paired *t*-test.

	*t*-test			*F* test to compare variances		
		
Parameter	*t*, *df*	*p*	Corrected *p*	*F*, DFn, Dfd	*p*	Corrected *p*
D	*t* = 0.8546, *df* = 335	0.3934	0.6865	1.166, 169, 166	0.3213	0.4988
SD_*D*_	*t* = 0.2732, *df* = 319	0.7849	0.9593	1.132, 166, 153	0.4350	0.5336
V	*t* = 0.8508, *df* = 335	0.3955	0.6865	1.168, 169, 166	0.3167	0.4988
SD_*V*_	*t* = 0.7785, *df* = 311	0.4369	0.6865	1.044, 167, 144	0.7934	0.7924
A	*t* = 0.09389, *df* = 225	0.9253	0.9895	4.276, 131, 94	**<0.0001**	**<0.001**
SD_*A*_	*t* = 0.01313, *df* = 327	0.9895	0.9895	1.153, 159, 168	0.3631	0.4988
M	*t* = 2.085, *df* = 341	**0.0378**	0.208	2.157, 167, 174	**<0.0001**	**<0.001**
SD_*M*_	*t* = 0.4351, *df* = 347	0.6638	0.9127	1.154, 171, 176	0.3466	0.4988
Ω	*t* = 7.435, *df* = 342	**<0.0001**	**<0.001**	1.106, 173, 169	0.5095	0.5624
SD_Ω_	*t* = 1.031, *df* = 349	0.3033	0.6865	1.486, 175, 174	**0.0092**	**0.0254**
PI	*t* = 1.080, *df* = 335	0.2809	0.6865	3.561, 167, 168	**<0.0001**	**<0.001**

*Tested “parameters” are swimming behaviour parameters used in this study. p < 0.05, N = 167. Significant p-values are in bold.*

**TABLE 3:** Statistical details for Repeated Measure (RM) Two-Way ANOVA with Geisser-Grenhouse Corrections.

Parameter	*F*_*time of day*_ (DFn, DFd)	*p*_*time of day*_	*F*_*phase*_	*p*_*phase*_	*F*_*interaction*_	*p*_*interaction*_
D	(1, 333) = 0.6796	=0.4103	(1, 333) = 285.4	**<0.0001**	(1, 333) = 7.981	**=0.0050**
SD_*D*_	(1, 317) = 2.156	=0.1431	(1, 317) = 259.8	**<0.0001**	(1, 317) = 4.796	**=0.0293**
V	(1, 333) = 0.6736	=0.4124	(1, 333) = 285.1	**<0.0001**	(1, 333) = 7.936	**=0.0051**
SD_*V*_	(1, 309) = 1.265	=0.2615	(1, 309) = 266.4	**<0.0001**	(1, 309) = 3.792	=0.0524
A	(1, 312) = 4.523	=**0.0342**	(1, 312) = 17.09	**<0.0001**	(1, 312) = 6.825	**=0.0094**
SD_*A*_	(1, 325) = 0.0017	=0.9670	(1, 325) = 28.67	**<0.0001**	(1, 325) = 0.5126	=0.4745
M	(1, 345) = 5.315	=**0.0217**	(1, 345) = 0.0948	=0.7583	(1, 345) = 2.884	=0.0903
SD_*M*_	(1, 345) = 0.1343	=0.7142	(1, 345) = 10.91	**=0.0011**	(1, 345) = 0.9228	=0.3374
Ω	(1, 340) = 23.28	<**0.0001**	(1, 340) = 0.3568	=0.5507	(1, 340) = 17.42	**<0.0001**
SD_Ω_	(1, 347) = 0.0599	=0.8068	(1, 347) = 11.24	**=0.0009**	(1, 347) = 0.3833	=0.5362
PI	(1, 333) = 3.302	=0.0701	(1, 333) = 216361	**<0.0001**	(1, 333) = 0.2985	=0.5852

*Factors are “time of the day” of hatching time, i.e., nighttime or daytime, and “phase” of the light dark challenge essay, i.e., light phase or dark phase. Tested “parameters” are swimming behaviour parameters used in this study. p < 0.05, N = 167. Significant p-values are in bold.*

**FIGURE 6:**
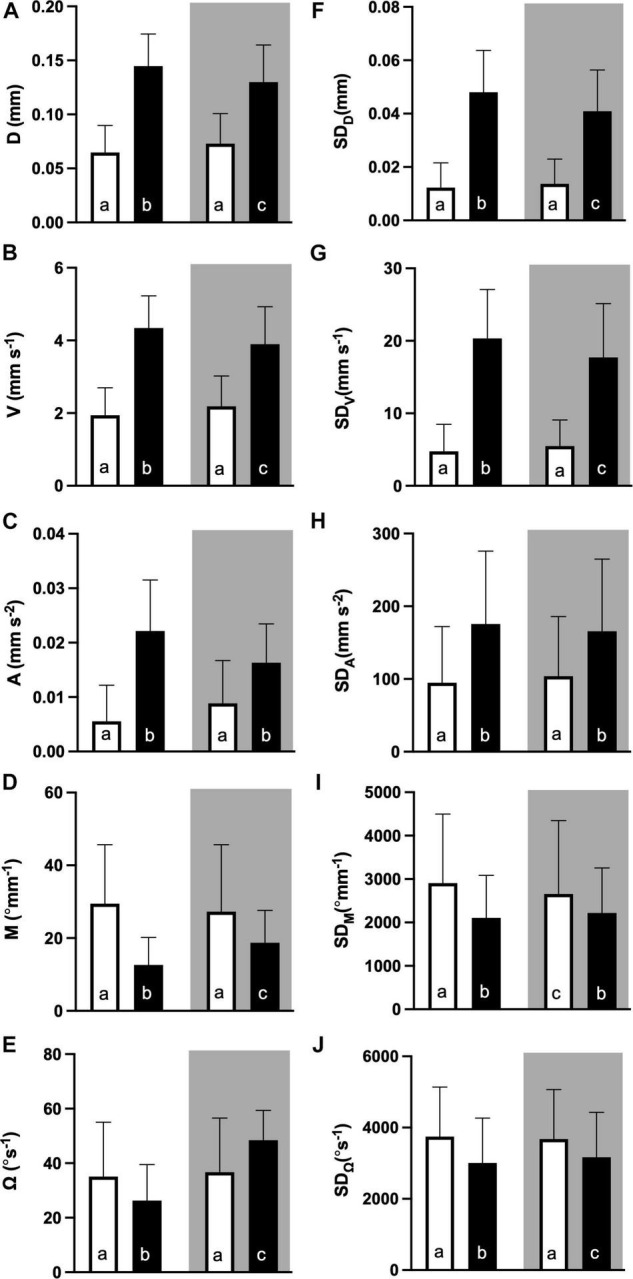
Parameters for swimming behaviour during the light dark challenge assay: **(A)** distance moved (D, mm), **(B)** velocity (V mm s^–1^), **(C)** acceleration (A mm s^–2^), **(D)** meandering (M, °mm^–1^), and **(E)** angular velocity (Ω, °s^–1^), and the variation thereof in standard deviation (SD, **F–J**), for the light (white bars) and dark (black bars) challenge phase of the essay. Shading indicates hatching during night time (dark shaded) and day time (not shaded). All values are mean ± SE. Letters indicate significant difference (RM Two-way ANOVA with Geisser-Greenhouse corrections, time of the day and phase as factors, Sidack’s *post hoc* test, *p* < 0.05, *N* = 167).

Sidac *post hoc* tests were used to identify differences between the values of time of day and phase (Figure 6). For all locomotion parameters, values reached during the dark phase of the light dark challenge assay were higher for individuals hatched during daytime, compared to those hatched during nighttime. In contrast, dark phase values for directionality parameters were lower for individuals hatched during daytime, than during nighttime. Finally, light phase values were generally lower than dark phase values for locomotion parameters, and higher for directionality parameters, with the exception of Ω, for which individuals hatched during daytime showed higher values, and individuals hatched during night time showed lower values during the light than during the dark phase.

As an additional control in order to assess basic differences in locomotor capacity over HT, maximum values of all behavioural parameters were correlated with HT. There were no significant correlations detected (Spearman rank, *N* = 24, *p* > 0.05), indicating no differences in locomotor capacity over HTs of the individuals.

## Discussion

In this study we performed a detailed analysis of the light dark challenge behavioural paradigm in zebrafish larvae, where a baseline activity phase in ambient light is followed by a series of sudden alternating dark and light phases, which elicit an anxiety-like response (Steenbergen et al., 2011; Schnörr et al., 2012; Luchtenburg et al., 2019). We used automated tracking software to generate a number of biomechanical swimming parameters related to locomotion, i.e., distance moved, swimming velocity, acceleration, and percentage mobility, and directionality, i.e., angular velocity, and meandering of swimming path. We also calculated the variances of the respective parameters, as an indication for intra-individual variability. Using Principal Component Analysis (PCA) we established general correlations between these parameters and their variances for the determination of interindividual variation in movement patterns.

Principal component analysis (Figure 4) revealed no correlation of the parameters or their variances between the dark and the light phase of the light dark challenge assay. This result indicates that the behaviour in the light phase and the dark phase of an individual test are not related. However, high collinearity was observed between behavioural parameters within the same phase (Figure 5). The locomotion parameter distance moved (D) appeared be most highly correlated with the other locomotion parameters tested, while the directionality parameter angular velocity (Ω) showed the strongest correlation with other directionality parameters. Since observed behaviours and their interpretation appear to be species-specific (White et al., 2020), D and Ω can be used for general evaluation of coping styles in larval zebrafish.

An explanation for the lack of correlation between behavioural patterns during different phases of the light dark test, can be different internal states. Since acclimatisation occurs at ambient light, a first challenge phase of sudden darkness represents a changing and potentially dangerous situation. This dark shock typically induces an immediate anxiety-like response of elevated swimming velocity (Steenbergen et al., 2011; Ellis et al., 2012; Schnörr et al., 2012; Peng et al., 2016; Luchtenburg et al., 2019). It was repeatedly assumed that upon acclimatisation in light the larva enters an anxiety-like state during the dark phase, while a reduced activity during the following light phase represents recovery back to baseline behaviour (Steenbergen et al., 2011; Schnörr et al., 2012). This assumption was supported by the finding that adding diazepam, an anxiolytic, reduces the swimming speed and adding caffeine, an anxiogenic, increases the swimming speed during the dark phase (Schnörr et al., 2012). However, Faught and Vijayan (2021) showed that upon acclimatisation in darkness, swimming speed was completely cancelled during a sudden light phase and gradually increased again during a following dark phase. The authors interpreted this phenomenon as a freezing bout during the light phase and recovery back to baseline swimming behaviour during the dark phase, and therefore questioning the paradigm of a standalone anxiety-like response to sudden darkness. We therefore propose a novel interpretation of the light dark paradigm that a sudden change in illumination elicits the anxiety-like response, regardless the acclimation and challenge conditions. Further pharmacological studies should confirm our suggestion.

We then tested the hypothesis that individual hatching time (HT) is correlated with behavioural parameters measured during the light dark paradigm. In order to test only for the diel light effect on HT, ambient temperature was kept constant. Hatching time in hours post-fertilisation (hpf), an absolute measure, showed no correlation with any of the mobility parameters. However, when transforming HT into Zeitgeber time (hZT), a significant correlation emerged only with the directionality parameters, i.e., meandering (M) and angular velocity (Ω), during the dark phase. Also, the amplitudes of a superimposed sinusoidal curve differed only for the directionality parameters between the phase of the behavioural assay, with higher values for dark than for the light phase. Moreover, when separating locomotion patterns of individuals hatched during daytime from those of individuals hatched during nighttime, the results showed a clear trend: day hatched individuals had higher values of movement related parameters D and V, and lower values of directionality M and Ω, during the dark phase of the behavioural test than night hatched individuals. Higher directionality levels have previously been deemed “erratic” swimming behaviour (Tudorache et al., 2015; Jaikumar et al., 2020), and their occurrence especially during the dark phase of the light dark challenge can be interpreted as an reflection of the anxiety-like state the larvae are in. This means that larvae hatched during nighttime show a stronger response to the dark phase of the light dark challenge, than larvae hatched during daytime. Interestingly, behavioural parameters during light phase did not differ with the time of the day they hatched, leading to the interpretation that individual variation of diel hatching time relates only to certain behavioural states.

As with many other correlated parameters within a behavioural syndrome, it is difficult to disentangle cause and effect and the question arises whether hatching time is responsible for behavioural output or that the behavioural phenotype gives rise to different hatching patterns, possibly through a difference in activity. It has been shown that high baseline activity individuals recovered slower from an acute stressor (Tudorache et al., 2015), and had a later chronotype (Amin et al., 2016) than individuals with a lower baseline activity. However, in rainbow trout, individuals which emerged earlier from spawning nests had a generally more proactive coping style than individuals which emerged later (Metcalfe and Thorpe, 1992; Andersson et al., 2013). This, together with our finding that daytime hatching individuals have a stronger anxiety-like response upon a dark challenge, suggests that coping style and hatching time are related in multiple dimensions within a behavioural syndrome.

## Data Availability Statement

The original contributions presented in the study are included in the article/Supplementary Material, further inquiries can be directed to the corresponding author.

## Ethics Statement

Ethical review and approval was not required for the animal study because Larval zebrafish younger than 6 dpf are not considered as experimental animals.

## Author Contributions

SR designed and performed the experiments, and analysed the data. FR analysed the data. MS designed the experiments. CT designed the experiments and analysed the data. All authors wrote the manuscript, contributed to the article, and approved the submitted version.

## Conflict of Interest

The authors declare that the research was conducted in the absence of any commercial or financial relationships that could be construed as a potential conflict of interest.

## Publisher’s Note

All claims expressed in this article are solely those of the authors and do not necessarily represent those of their affiliated organizations, or those of the publisher, the editors and the reviewers. Any product that may be evaluated in this article, or claim that may be made by its manufacturer, is not guaranteed or endorsed by the publisher.

## Footnotes

1
http://ZFIN.org
