# Supplementary material for: Detailed Analysis of Zebrafish Larval Behaviour in the Light Dark Challenge Assay Shows That Diel Hatching Time Determines Individual Variation
Source: Front Physiol. 2022 Apr 11;13:827282. doi: 10.3389/fphys.2022.827282 (PMC9036179; doi:10.3389/fphys.2022.827282)
Supplement: Supplementary file 1 [file Data_Sheet_1.docx]

Supplementary Material

# Supplementary Figure 1. The relationship between HT and SD(M)_dark_ after accounting for their linear association with all other variables. The solid blue line shows the estimated partial correlation the dashed lines a 95% confidence interval and the dotted lines a 95% prediction interval. (Spearman rank, ρ_partial_ = -0.17, p=0.0281).

**Supplemental figure 2:** Raw data of the light and the dark phase parameters for swimming behaviour during the light dark challenge assay: A) distance moved (D, mm), B) velocity (V mm s^-1^), C) acceleration (A mm s^-2^), D) meandering (M, °mm^-1^), and E) angular velocity (Ω, °s^-1^ ), and the variation thereof in standard deviation (SD; F-J), for the light (white, grey) and dark (black) challenge phase of the essay. A sine wave (non-zero baseline, phase = 24 hours, dotted) and a linear regression (continuous) were fitted over the data points. Regression values are given for the light (white, grey) and dark (black) challenge phase (Spearman rank, p<0.05, N=167).

**Supplemental figure 3:** Amplitudes of a sine wave (non-zero baseline, phase = 24 hours) which was fitted over the data points of the light and the dark phase parameters for swimming behaviour during the light dark challenge assay: A) distance moved (D, mm), B) velocity (V mm s^-1^), C) acceleration (A mm s^-2^), D) meandering (M, °mm^-1^), and E) angular velocity (Ω, °s^-1^ ), and the variation thereof in standard deviation (SD; F-J), for the light (white) and dark (black) challenge phase of the essay. All values are mean ± SE. Stars indicate significant difference (student t-test, p<0.05, N=167).
